# Individual risk preference as a predictor of health behaviour: evidence from the use of condoms against HIV/AIDS in Ghana

**DOI:** 10.1186/s12889-023-16579-7

**Published:** 2023-08-29

**Authors:** Abel Gbogbolu, Edward Nketiah-Amponsah

**Affiliations:** Department of Economics, University of Ghana, Legon, Accra, Ghana

**Keywords:** Risk preference, Risk-averse, Risk-lover, Condom use, HIV/AIDS, Ghana

## Abstract

**Background:**

It is evident that public health education interventions to promote the use of condoms against HIV infections in Ghana have yielded modest results. However, existing studies in the field of sexual and reproductive health in Ghana have failed to account for differences in risk preferences of individuals. This study fills the gap by investigating how individuals' risk preferences predict their behaviour toward using condoms against HIV in Ghana.

**Method:**

Conceptually, the study followed the Grossman health capital theoretical model for risk preference and health behaviour nexus. Data were obtained from the most recent Ghana Living Standards Survey Round 7 (GLSS 7), conducted in 2017. Using data from GLSS 7, a probit regression model was estimated to show how the risk preferences of individuals that did not abstain from sex predicted their use of condoms against HIV. To ensure robustness, two scenarios of declared risk preferences were used to predict the use of condom behaviour against HIV.

**Results:**

Probit regression estimation shows that the risk preferences of individuals that did not abstain from sex significantly predicted their use of condoms against HIV in Ghana. Even though the study found that the predicted probability of using a condom reduces among risk-averse individuals that do not abstain from sex, not using a condom against HIV was found to be worse among risk lovers.

**Conclusion:**

The study provides empirical evidence that public health education against HIV/AIDS in Ghana cannot continue to ignore the risk preference of individuals. The results of this study have immediate implications, first for a comprehensive and continuous measurement of risk preferences among Ghanaians in major household surveys going forward. At the moment, the latest round of the GLSS is just about the only household survey in Ghana that has attempted to collect some data on individual time and risk preferences using only hypothetical monetary rewards. Second is the immediate consideration of individual risk preferences in public health education campaigns against HIV/AIDS in Ghana.

## Background

Sub-Saharan Africa (SSA) had the highest incidence of human immunodeficiency virus/acquired immunodeficiency syndrome (HIV/AIDS) globally in 2015, when developing countries were expected to have met the millennium development goals (MDGs) on HIV/AIDS [1]. The Sustainable Development Goals (SDGs) and more specifically SDG-3 were thus introduced as a follow-up to the MDGs to reduce the annual infection incident rates of HIV/AIDS by 70% by the end of 2030 [2]. The World Health Organisation [3] estimates that the SSA region has a prevalence rate of 3.6%, accounting for about two-thirds of all HIV-positive individuals globally. Persons between the ages of 15 and 49 years in SSA have the greatest incidence of HIV worldwide; the highest incidences emanating from South Africa (19.1%), Botswana (19.9%), Eswatini (26.8%) and Lesotho (21.1%) [3]. Consequently, by 2025, UNAIDS estimated that low- and middle-income countries (LMICs) – mainly SSA – would need to spend roughly USD30 billion to successfully eradicate HIV/AIDS [3].

The HIV prevalence in Ghana, which is 1.7%, is among the relatively lower rates in SSA [4], a decline from 2% prevalence reported in 2014 [5]. Despite the drop from 2% to 1.7%, the disease still presents serious consequences for health systems and the economy [6]. According to the Ghana Aids Commission [7], there are approximately 13,616 annual HIV/AIDS-related deaths in Ghana, and among young people aged 15 to 24 years, the prevalence of HIV has increased. The country's attempts to eliminate HIV/AIDS as a public health concern by 2030 (SDG 3, goal 3.3) have been cited as being threatened by the prevalence among this young age group [8].

HIV/AIDS still has no known treatment; hence, the only societal immunisation against the illness is education on preventive measures. Here, education refers to everything that is done to raise and maintain people's HIV/AIDS awareness and understanding in order to discourage risky behaviours. A phenomenon that is however often ignored in this equation but remains important in understanding health behaviours is the risk preference of individuals. Risk preference of individuals has been suggested to influence health decision-making and risky behaviour [9–12]. Health decision-making may involve the utilisation of preventative medical care and lifestyle tendency to engage in dangerous sexual behaviours, cigarette smoking, or even seat belt use, all of which have morbidity and mortality risks [11].

The way individuals make decisions involving uncertainty and risk and their impact on health-related behaviours have been studied by few scholars with important implications for health policy. Fuchs [9], Holt and Laury [10], Anderson and Mellor [11], Herberholz [13], among others, suggest that risk-averse individuals are more likely to avoid harmful health-related behaviours such as risky sexual behaviours. Generally, risky sexual behaviour is described as any sexual behaviour that predisposes a person to risk sexually transmitted infections (STIs) like HIV and unintended pregnancy [14, 15]. Thus, the use of contraceptives and condoms in particular either reduces the risk of STIs such as HIV/AIDS and/or unintended pregnancy.

Intrinsic to risky sexual behaviours and the use of contraceptives against STIs is the risk preference of the individual [16]. Risk preference has been considered from both psychological and monetary perspectives in health economics. Risk preference in psychology is generally defined as the predisposition to participate in activities that, while rewarding, carry the risk of damage or loss [17]. Risk preference in economics and finance describes the propensity to choose a probable monetary outcome from an activity with a higher degree of uncertainty than one with a lower degree of uncertainty, given same expected value [18].

Existing studies on the phenomenon have neglected the particular effect of individual risk preferences on adopting and using contraceptives (condoms) against STIs, particularly HIV/AIDS. Many HIV/AIDS health policies of countries are yet to recognise the importation of individual traits in formulating and implementing their 'social immunisation' campaigns. This study fills the gap by investigating how the risk preferences of sexually active Ghanaians predict their behaviour toward using condoms against HIV. This study is novel in that it is the first to engage the already estimated hypothetical gamble reward questions as a measure of risk preference following Binswanger [19] in the Ghana Living Standards Survey (GLSS) round 7. This is used as an interest predictor variable to predict the use of condoms for HIV protection in Ghana.

## Literature review

### Conceptual underpinning

This study is inspired by Grossman's health capital model, which proposes that individuals augment depletions in their inherited health stock by investing in health, adopting and avoiding (un)healthy behaviours such that over their lifetimes, their expected discounted utility is maximised [20, 21]. In the Grossman model, time preferences influence the discount rate at which future health-promoting behaviours are discounted [22]. Individuals with higher discount rates are expected to invest less in their health and engage in more unhealthy or risk behaviours.

However, risk preference was assumed away from the model since Grossman's model was formulated under conditions of uncertainty. Uncertainty, which opened the door for risk preference impact analyses, was added to the model by Dardanoni and Wagstaff [23]. Pfeifer [24] investigated how variations in risk preferences affected a basic Grossman model. The conclusions are that risk preferences impact health investments and productivity in health. Risk-averse individuals are expected to invest more heavily in their health than risk lovers do to reduce their risks, demonstrating their readiness to embrace more uncertainty in the course of their health [24]. Evidence from recent studies, therefore, shows a connection between risk preferences and health behaviours [25, 26].

The use of condoms against HIV enters the model since investments in health are also affected by expected health consequences. Evidence shows that most people underestimate the likelihood and degree of the negative health effects of unhealthy behaviours, and, as a result, underestimate the benefits of making investments in health prevention [27].

### The use of condoms in Ghana

Condoms have been universally acknowledged as a vital component in global efforts to prevent and manage sexually transmitted infections (STIs). Several authors have demonstrated the usefulness of condoms in decreasing the spread of HIV and other STIs when worn regularly and appropriately [28, 29]. Health experts and policymakers have expanded their efforts to promote condom usage among diverse communities in order to prevent STIs and unplanned births. Condoms have played a critical role in reducing HIV transmission and controlling the disease's spread in regions where the virus is prevalent. For example, policy programs aimed at distributing condoms to diverse groups have been demonstrated to lower HIV among gays and sex workers [30, 31].

In Ghana, attempts have been made to increase condom use among young people. Some of these include providing youth-friendly centers to give complete education on sexual health, which also improves access to services like condoms at a low cost for young people, and promoting condom usage through media and community outreach initiatives [32]. Despite these initiatives to support good sexual and reproductive health, including greater knowledge of condoms' efficacy in preventing STIs, their usage among adolescents and young adults in Ghana is still low and irregular [33]. The national prevalence and use of contraception mainly stand at about 18.6% among sexually active young adults [34]. Karim et al*.* [35], in their cross-sectional survey, found that only 18% of unmarried males in Ghana used condoms during their first sexual encounter.

Researchers that have sought to explain the causes of Ghanaian adults' poor and irregular condom use have mainly concentrated on specific demographics such as sex workers. Most studies in Ghana have focused on examining aggregate attitudes, beliefs, and the frequency of particular risk behaviours across contexts and locations [36]. Studies in the field of sexual and reproductive health in Ghana have not considered the variation of risk preferences as predictors of certain behaviour in a given situation [37]. This study is, however, based on the Grossman health capital theoretical model as modified for risk preference by Dardanoni and Wagstaff [23] and further verified by Pfeifer [24] to investigate the predictive influence of risk preferences.

### Risk preference and health-related behaviour

A core argument in the risk preference-health behaviour nexus is whether an individual's level of evaluated risk preference could actually predict health behaviour. Numerous studies have examined risky behaviours under the assumption that they may be motivated by risk preferences in other contexts, such as financial risk [11, 26, 38], and they have found a positive correlation between financial risk tolerance and health-related behaviours, both for positive and negative health bahaviours.

Lepine and Treibich [25] examined the impact of risk preference on the sexual behaviours of female sex workers. They discovered that risk-averse sex workers are less likely to partake in unsafe sex and want more preventive services. Their results therefore align with those of other studies [11, 26, 38] that suggest risk preference could predict good health behaviour. Notwithstanding the significance of risk preference in these studies, individual risk preference in health decision-making and lifestyle could not be confirmed in other studies. According to a number of studies [39–42], there is either no direct relationship or a weak relationship between attitudes toward financial lottery and portfolio selection and health behaviour. Therefore, the results of empirical studies are divergent in context and specific outcomes of health behaviour, and the context under study in this study is a grey area that warrants investigation. Overall, the empirical literature lends credence to the idea that many risky health-related behaviours are linked to risk-tolerant preferences [43]. However, for health prevention behaviours, the foregoing reviews show that the predictive power of risk preferences may be weak or inconsistent. This study, therefore, provides an opportunity to test this relationship for Ghana.

## Methods

### Data

Data for this study was sourced from the seventh round of the Ghana Living Standards Survey (GLSS). The GLSS is a nationwide survey that collects data on a wide range of issues at the individual, household, and community levels. It is a household-based survey that focuses on key socioeconomic characteristics and the well-being of households in the country. The GLSS7 was conducted by the Ghana Statistical Service (GSS) from 22nd October 2016 to 17th October 2017. Five sets of questionnaires were used in the surveys: (1) a household questionnaire, (2) a non-farm household questionnaire, (3) a community questionnaire, (4) a governance, peace, and security questionnaire, and (5) a questionnaire about the price of food and non-food items. A stratified sampling design was implemented in two stages for data collection. In the first step, 1,000 Enumeration Areas (EAs), which served as the primary sampling units (PSUs), were randomly chosen from the entire nation to provide a nationally representative sample. With the aid of stratified systematic probability proportionate to size (PPS), the PSUs were distributed across the ten areas. In addition, the EAs were split into 562 rural localities of residence and 438 urban areas. A complete listing of households in the selected PSUs was undertaken to form the secondary sampling units (SSUs). The second stage involved conducting a household listing operation in all of the designated EAs nationally, where 15 households from each SSU were systematically chosen, resulting in a total sample of 15,000 households nationwide. With a sample size of 15,000 households chosen from 1,000 enumeration areas, the survey is nationally representative. The survey received responses from 14,009 households in total, yielding a 93.3 percent response rate. Detailed information was collected on Demographic characteristics; education, health, employment, migration, tourism, housing, household agriculture, expenditure and income, governance, peace and security, financial services, credit, and assets. For the purposes of this study and in line with our objectives, the relevant data captured in the GLSS7 included information from the already estimated hypothetical gamble reward questions as a measure of risk preference following Binswanger [19], contraceptive (condom) use among individuals 18 years and above, who responded to the contraceptive use questionnaire. Other relevant socio-demographic control variables of the individuals outlined in Table 1 were also extracted from the GLSS7.

**Table 1:** Description of variables

Variable	Operationalisation
*Outcome variable*	
Condom use (CD_i_)	1 if individual used condom, 0 otherwise
*Interest predictor variables*	
Risk preference	1if individual is risk averse; 0 otherwise
Risk preference # abstain interaction	1 individual risk preference if he abstains from sex, 0 individual risk preference if he does not abstain
*Control variables*	
Age	Age of individual (18 years and above)
Gender	1 male, 0 female
Religion	Religion of individual
Educational status	1 educated, 0 uneducated
Employment status	1 if individual is employed; 0 otherwise
Locality	1 if individual is in urban, 0 rural
Poverty status	1 if individual is poor, 0 non-poor
Sexual abstinence	1 Individual do not abstain, 0 abstain

Source: Ghana Living Standards Survey Round 7

After merging and cleaning the datasets of the relevant variables from the GLSS7, a total sample of 10,252 and 10,251 observations were used in the analyses under both scenarios. A summary overview of all the relevant variables extracted is summarized in Table 2.

**Table 2:** A summary overview of extracted variables

Variable	Obs	Mean	Std. Dev	Min	Max
Condom use	10,252	0.542	0.498	0	1
Risk preference	10,252	0.574	0.495	0	1
Age (years)	10,252	30.03	18.52	18	90
Gender	10,252	0.688	0.464	0	1
Educational status	10,252	0.381	0.486	0	1
Locality	10,252	0.430	0.476	0	1
Poverty status	10,252	0.879	0.326	0	1
Employment status	10,252	0.221	0.415	0	1
Sexual abstinence	10,252	0.608	0.488	0	1
Religion	10,252	2.200	0.519	1	4

Source: Ghana Living Standards Survey Round 7

### Measurement of risk preference

Risk preference is simply the way individuals make decisions involving uncertainty and risk [16]. In the expected utility framework, risk preference is operationalised as risk attitudes derived from people's choices. However, differences in risk attitudes are nothing more than a description of the individual utility function derived from a series of such choices [44]. Arrow [45] and Pratt [46] created a widely used measure for defining risk preference. They defined risk preference as the negative ratio between the second and first derivatives of the utility function. Choosing a fixed (sure) sum of money over a lottery with equal anticipated value indicates a risk-averse (concave) utility function, whereas choosing the lottery indicates a risk-seeking (convex) utility function, and indifference indicates a risk-neutral (linear) utility function.

Risk preference in this study is measured by following the ordered lottery selection version of Binswanger [19] as already estimated in the GLSS7. This procedure is a hypothetical reward gamble between a certainty equivalent and a lottery [47]. The lottery has a 50% chance of occurring and differing in anticipated return and volatility. In the GLSS7, two lotteries differing in anticipated higher returns at 50–50 chance were compared with certainty equivalents (see Table 3). Risk-averse individuals were expected to choose certainty equivalents, while risk-loving individuals chose the lottery. This study is constrained to measure risk aversion as a binary variable as opposed to an ordinal measure with different levels of risk aversion due to the nature of the question asked in the survey. Studies such as Frempong and Stadelmann [48], Adjei-Mantey and Horioka [49], Adjei-Mantey and Takeuchi [50] used a similar measure of risk preference. The GLSS7 particularly used binary lottery scenarios instead of experiments to avoid making assumptions about the utility functions of the individuals.

**Table 3:** Hypothetical reward gamble measure of risk preference

Risk preference	Option 1	Option 2
Scenario 1	a) You receive 4 Ghana Cedis for sure	b) I flip a 1 Cedi Coin. If it shows the Shell, you get 12 Ghana Cedis. If it's the coat of arms, you get 1 Ghana Cedi
Scenario 2	c) You receive 4 Ghana Cedis for sure	d) I flip a 1 GH Cedi Coin. If it shows the Shell, you get 16 Ghana Cedis. If it's the coat of arms, you get 1 Ghana Cedi

Source: Ghana Living Standards Survey Round 7

It is important to note that as is customary in these surveys, the hypothetical lotteries do not either benefit or loss to participants [49]; as a result, the response may not accurately reflect the participants actual risk tolerance. However, research that have looked at how self-reported relates to real risk preferences revealed significant correlations and consistency between the two measures [19, 50].

In scenario 1, the respondents were asked to choose between hypothetical options (a) and (b). In Scenario 2 also, the respondents had to choose between hypothetical options (c) and (d). Respondents' choices in (a) and (c) represent risk-averse preference behaviour while (b) and (d) represent risk-loving preference behaviour. The relatively higher hypothetical reward in (d) compared to (b) was meant to elicit whether risk-loving choices increases with higher reward. The GLSS7 in framing the risk preference questions for the national observational survey basically purposed to see if an effect can be ascertained on hypothetical rewards of relatively small magnitudes as much as possible on binary levels.

### Empirical model and estimation

The outcome variable of interest is whether an individual will use condom to protect himself from HIV. That decision is a latent trait, unobservable until the event that an individual uses a condom for that purpose. The effect of individual* i* risk preference (Riskpref) on his decision to use condom against HIV can be defined as

1 $${CD}_{i}={{x}{\prime}}_{i}{a}_{i}+{Riskpref{\prime}}_{i}{\beta }_{i}+ {Abstn}_{i}* {Riskpref{\prime}}_{i}{n}_{i}+{u}_{i}$$

where *CD*_*i*_ is the outcome condom use variable categorised according to 1 'use' and 0 'non-use', *x*_*i*_ is a vector of explanatory control variables, *Riskpref*_*i*_ is risk preference of individual *i* (1, an individual is risk averse or 0 risk lover), *α*_*i*_,* β*_*i*_, *η*_*i*_ are vectors of parameters to be estimated, *u*_*i*_ is the random error term. *Abstn* is whether individual* i* abstains from sex and Abstn*Riskpref directly captures the risk preference of individuals that did not abstain from sex.

Primarily, individuals who totally abstain from sex are excluded from the analyses and must be accounted for in the risk preference variable. Since abstain (Abstn), and risk preference are both binary, an interaction of both variables allows us to accurately measure the risk preferences of individuals that did not abstain from sex. Hence, the riskpreference**#**abstain interaction term enters the model for study (Table 1).

The outcome *CD*_*i*_ status variable is binary given by the relationship

$$\begin{array}{l}{CD}_{i} = 1 if {CD}_{i} > 0 (\mathrm{individual\;used\;condom})\\ {CD}_{i} = 0\mathrm{ if }{CD}_{i} < 0(\mathrm{individual\;did\;not\;use\;condom})\end{array}$$

The probability that an individual will use a condom against HIV given his risk preference behaviour and other covariate socio-demographic characteristics is obtained by

2 $$Pr({CD}_{i} =1|{x}_{i},{Riskpref}_{i} ) = Pr({CD}_{i} > 0) = Pr({x{\prime}}_{i}{\alpha }_{i} + R{iskpref{\prime}}_{i}{\beta }_{i}+{ Abstn}_{i}*{Riskpref{\prime}}_{i}{\eta }_{i} +{u}_{i}) = \phi ({x{\prime}}_{i}{\alpha }_{i}+ {Riskpref{\prime}}_{i}{\beta }_{i} + {Abstn}_{i}*{Riskpref{\prime}}_{i}{\eta }_{i}),$$

where ɸ is the cumulative distribution function of the standard normal distribution. A probit regression estimation is used to estimate Eq. 1, and the results are discussed below.

## Results

### Descriptive analysis

The descriptive summary of the outcome variable, condom use behaviour against HIV, was considered for the two 50–50 hypothetical risk preference scenarios in the GLSS7, as summarised in Table 2. Therefore two regression models were generated for the scenarios for robustness comparison of results. The descriptive statistics for both scenarios are summarised in Table 3 for the condom use behaviour outcome variable.

Risk preferences 1 and 2 for both gambles chosen bu the respondents show no marginal difference in the total preferences made. While a total of 10,252 individuals chose risk preference 1, a total of 10,251 chose risk preference 2. Perhaps because most of the respondents captured in the GLSS7 were risk averse by attitude, it makes no difference choosing (a) in scenario 1 and (c) in scenario 2. Total risk-averse individuals in scenario 1, representing 76.76% differed not much from a total of 76.06% recorded for scenario 2.

**Fig. 1:**
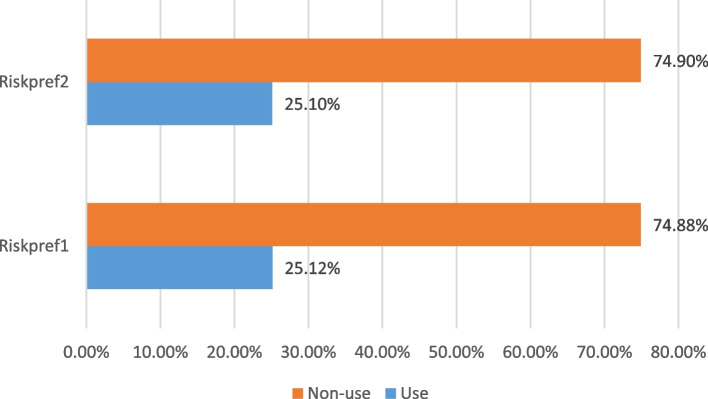
Condom use behaviour according to risk preference scenarios. Source: Author's construct from GLSS7

In terms of recorded numbers, risk preference percentages were not much different across condom use behaviour against HIV (Fig. 1). The results summarised in Fig. 1 show that for both risk preference scenarios, while a total of 25% individuals will use condom against HIV, a total of about 75% will not. For those that will not use condom, a total of 57.96% and 57.35% were risk averse for risk preference scenarios 1 and 2 respectively, while 16.92% and 17.55% were respectively risk loving (Table 4). On the other hand, a total of 18.80% and 18.71% were risk averse for those that will use condom respectively for preference scenarios 1 and 2. The risk lovers that will use condom to protect themselves against HIV were only about 6% for both risk preference scenarios.

**Table 4:** Descriptive summaries of condom use on hypothetical risk preference choices

If condom use	Risk preference 1			Risk preference 2		
		Count	Percent		Count	Percent
Non-use	Risk lover	1,735	16.92%	Risk lover	1,799	17.55%
	Risk averse	5,942	57.96%	Risk averse	5,879	57.35%
Use	Risk lover	648	6.32%	Risk lover	655	6.39%
	Risk averse	1,927	18.80%	Risk averse	1,918	18.71%
Total		10,252	100%		10,251	100%

Source: Author's construct from GLSS7

The results appear to suggest that risk-averse individuals will use condom more than risk-loving individuals to protect themselves against HIV. This is consistent with some empirical literature [11, 25] depending on the final results of analyses for this paper (Table 6). However, those that will not use condom for protection, the majority being risk averse, are theoretically expected to resort to other means (presumed safer) for protection. One such means found to highly correlate with the condom use outcome variable and of interest to the predictor variables is whether or not the individual abstains from sex.

Intuitively, the risk preferences of individuals who totally abstain from sex are of no relevance to the analyses of this paper. However, the predictor risk preferences binary variable described in Table 2 crudely includes individuals who abstain from sex. From Table 5, a total of about 32% consist of all risk preferences of individuals who totally abstained from sex; of course, the majority being risk averse individuals of about 25% for both risk preference scenarios (Table 5). Therefore, the inclusion of an interaction between the risk preferences and abstain binary variables addresses this issue. The interaction term enters the model such that only risk preference behaviours of individuals that did not abstain from sex are investigated (Table 6).

**Table 5:** Descriptives of if abstain from sex on hypothetical risk preference choices

Abstain	Risk preference 1			Risk preference 2		
		Count	Percent		Count	Percent
No	Risk lover	1,602	15.63%	Risk lover	1,647	16.07%
	Risk averse	5,333	52.02%	Risk averse	5,287	51.58%
Yes	Risk lover	781	7.62%	Risk lover	807	7.87%
	Risk averse	2,536	24.74%	Risk averse	2,510	24.49%
Total		10,252	100%		10,251	100%

Source: Author's construct from GLSS7

**Table 6:** Probit regression results: condom use behaviour against HIV

	(1)	(2)
Variables	Condom use	Condom use
Gender (ref female)		
*Male*	0.168*** [0.0491]	0.168***[0.0492]
	(0.0320)	(0.0320)
Age(Yrs)	-0.0115***[-0.0035]	-0.0116***[-0.0035]
	(0.000933)	(0.000933)
Location (ref rural)		
*Urban*	0.129***[-0.0388]	0.129***[-0.0388]
	(0.0300)	(0.0300)
Religion (ref no religion)		
*Christian*	-0.0386[-0.0119]	-0.0378[-0.0117]
	(0.0610)	(0.0610)
*Islam*	-0.202***[-0.0593]	-0.202***[0.0591]
	(0.0679)	(0.0679)
*Traditional*	-0.164**[-0.0488]	-0.163*[0.0482]
	(0.0833)	(0.0833)
Education status (ref uneducated)		
*Educated*	0.121***[0.0355]	0.121***[0.0354]
	(0.0390)	(0.0390)
Employment status (ref employed)		
*Unemployed*	-0.101***[-0.0301]	-0.100***[ -0.0299]
	(0.0288)	(0.0288)
Poverty status (ref non-poor)		
*Poor*	0.314***[0.0858]	0.315***[0.0861]
	(0.0574)	(0.0574)
Risk preference (ref risk averse)		
*Risk lover*	-0.0510[ -0.0177]	-0.0497[0.0104]
	(0.0539)	(0.0533)
Riskpreference#Abstain (ref Yes/Abstain)		
*Risklover#No/abstain*	-0.469***[-0.1348]	-0.46***[-0.1347]
	(0.0333)	(0.0335)
*Riskaverse#No/abstain*	-0.295***[-0.0471]	-0.326***[-0.0388]
	(0.0589)	(0.0582)
Constant	-0.162	-0.162
	(0.107)	(0.107)
No. of obs	10,252	10,251
LR chi2(12)	694.92	690.59
Prob > chi2	0.0000	0.0000
Pseudo R2	0.0603	0.06

Standard errors in parentheses ()Marginal effects in adjacent brackets []

^***^ *p* < 0.01, ** *p* < 0.05, * *p* < 0.1

Source: Author's estimations from GLSS7

### How risk preference predicted the use of condom against HIV

The probit regression estimation results as well as marginal effects for the model equation (i), based on the two hypothetical scenarios are summarised in Table. The total observations used for the two estimated models are 10,252 and 10,251 respectively. The likelihood ratio (LR) chi-square test that at least one of the regression coefficients in the model is not equal to zero is highly significant at 1%.

The results show that for both risk preference scenarios (i.e. both model 1 and 2), switching from risk averse to risk lover is associated with a reduced predicted probability of using condom to protect against HIV. This suggests a negative relationship between risk lovers and using condom for STI protection. The results are however insignificant (*β*_*1*_ = *-0.051, p*_*1*_ = *0.344* and *β*_*2*_ = -0*.04966, p*_*2*_ = *0.352*) (Table 6). Grossly, these results seemingly agree with other risk preference and health prevention behaviour studies suggesting a weak relationship between the nexus. Love and Smith [39], Fan and Shao [40], Cardak and Wilkins [42], among others, have found either no direct relationship or weak relationship between attitude to financial risk preference and health prevention behaviour.

However, when the risk preferences of individuals who totally abstained from sex were accounted for, the results indicate that the interaction of risk preferences and abstinence variable (Riskpreference#Abstain) have significant associationwith condom use in two but same directions. First, the results show that for individuals that did not abstain from sex, switching from risk averse to risk lover significantly reduces the probability of using condom to protect against HIV in both risk preference scenarios at 1% level (*β*_*1*_ = *-0.4693754, β*_*2*_ = *-0.46026. p* = *0.000*). (See the coefficient of *Risklover#No/abstain* interaction term in Table 6). These results are strengthened by the marginal effects of -0.1348 and -0.1347, respectively, suggesting a negative effect relationship between risk-loving attitude (accounted for sex abstinence) and condom use against HIV.

These findings are in line with de Oliveira et al. [12], Anderson and Mellor [11] and Sutter et al*.* [51], Leonard et al*.* [52], Lepine and Treibich [25]. Anderson and Mellor [11] and Sutter et al*.* [51] found that individuals that are more risk-loving are more accommodating of future health consequences and may ignore immediate health prevention behaviours like using condom. de Oliveira et al*.* [12] also found a similar relationship in line with Anderson and Mellor [11] and Sutter et al*.* [49]. Second and surprisingly, after accounting for risk preferences of individuals that abstained from sex, the results also show that switching from risk lover to risk-averse significantly reduces the probability of using condom to protect against HIV in both models at 1% level (*β*_*1*_ = *-0.294981, β*_*2*_ = *-0.32585, p* = *0.000*). (See the coefficient of *Riskaverse#No/abstain* interaction term in Table 6). The estimated marginal effects of -0.0471 and -0.0388, respectively, suggest a significant predictive negative relationship between risk-averse attitude (accounted for sex abstinence) and condom use against HIV. On its own, this evidence seems to depart from a priori general findings that risk aversion preference promotes good health-related behaviour [25]. For instance, in Senegal, Lepine and Treibich [25], examining the impact of risk preference on sexual behaviours of female sex workers, discovered that risk-averse sex workers are less likely to partake in unsafe sex and will want more preventive services.

## Discussion

A few explanations are imperative for this new evidence within the context of the Ghanaian environment, which may be useful in situating the results within the empirical literature. First, we observe that the absolute marginal effects of *Risklover#No/abstain* are higher than those of *Riskaverse#No/abstain* (|*β*_*1*_|= *0.1348* > *0.0471,* |*β*_*2*_|= *0.1347* > *0.0388*). This suggests that for a higher switch in risk preference (from risk-averse to risk loving or from risk loving to risk-averse), we expect a relatively larger reduced probability of using condoms among risk-lovers than for risk-averse individuals. In other words, the degree of reductions in probability is higher in risk loving than risk-averse individuals, given that they are not abstaining from sex. Therefore, the picture this evidence paints – that if risk-averse individuals that participate in sex will not use condom then risk-loving individuals are even worse – fall in line with de Oliveira et al*.* [12], Anderson and Mellor [11], Sutter et al*.* [51] and even Lepine and Treibich [25].

Second, this study takes particular notice that de Oliveira et al*.* [12], Anderson and Mellor [11], Sutter et al*.*[51], among others, have focused on other health prevention behaviour outcomes away from a direct focus on condom use behaviour. These include physical activity, obesity, and medical checkup, among others. The closest was the work by Lepine and Treibich [25] in Senegal which did not also focus on condom use directly as an outcome variable. Therefore, the evidence generated in this paper may assume unique expressions in the particular focus of using condoms against HIV/AIDS as an outcome variable.

Third is the long-standing argument in the empirical literature that risk-averse individuals are less likely to be having indiscriminate sex or may be faithful to their partners [53] as a way of protecting themselves against HIV/AIDS. In this case, we expect to see a negative relationship between these risk-averse individuals and using condoms to prevent HIV/AIDS.

Beyond the predictor variables of interest, the effects of the following control variables are worth commenting on. Being educated significantly increases the predicted probability of using condom against HIV at 1% significance level. This is particularly important within the Grossman theoretical model for this paper that educated people are better producers of health. Being a male significantly increases the predicted probability of using condom against HIV at 1% significance level. This is consistent with apriori expectations. Individuals ageing in years reduces the predicted probability of using condom against HIV at 1% significance level. This is consistent with apriori expectations because as individuals age, their sexual adventure reduces and thus may reduce condom usage. Individuals living in rural areas reduces the predicted probability of using condom against HIV at 1% significance level. Being religious is also found to reduce the predicted probability of using condom against HIV, but only Islamic and Traditional religion are significant at 1% and 5% levels respectively. Being unemployed reduces the predicted probability of using condom against HIV at 1% significance level. Interestingly, it was found that the predicted probability of using condom against HIV increases among the poor than the non-poor at 1% significance level.

## Conclusion

It is evident from the literature that behavioural and educational interventions initiated to promote condom use against STDs and as a contraceptive in Ghana have only yielded modest results [33]. Against this backdrop, this study explored how individuals' risk preferences predict their behaviour toward using condoms against HIV in Ghana. The study followed the Grossman health capital theoretical model as modified for risk preference by Dardanoni and Wagstaff [23] and further validated by Pfeifer [24]. Dardanoni and Wagstaff [23], Pfeifer [24] theorised that risk preferences affect the basic Grossman model such that risk-averse individuals, compared to risk-lovers, are less ready to embrace more uncertainty in the course of their health and are expected to invest heavily in good health-related behaviours. This therefore guided a quantitative modeling of risk preferences on condom use behaviour in Ghana.

Using data from the GLSS 7, a probit regression estimation of the model found that the risk preferences of individuals that did not abstain from sex significantly related to their use of condom to prevent HIV. Even though the study found that the probability of using condom reduces among risk-averse individuals that do not abstain from sex, the phenomenon was found to be worse among risk lovers.

The results of this study have immediate implications, first for a comprehensive and continuous measurement of risk preferences among Ghanaians in major household surveys going forward. At the moment, the latest round of the GLSS is just about the only household survey in Ghana that has attempted to collect some data on individual time and risk preferences using only hypothetical monetary rewards. It is suggested that the formats presented in the GLSS7 should be expanded to allow for more robust computation of the relative risk coefficients by researchers. Moreover, apart from monetary rewards, there are risk and temporal discounting measurements that consider hypothetical health rewards, and findings have shown differing results from when hypothetical monetary rewards are used. Therefore, the GLSS, GDHS and other household surveys must consistently track changes in individual risk and even time preferences, since this study has shown they could influence the success of public health interventions. Ghana and other SSA countries must begin to recognise the innate risk preferences of their populations in formulating and implementing their social vaccination and public health programs against HIV/AIDS.

## Data Availability

The dataset(s) supporting the analysis and conclusions of this study is (are) available at the Ghana Statistical Service Data repository. The data can can be retrieved from the official website of the Ghana Statistical Service (https://www.statsghana.gov.gh/gssdatadownloadspage.php).

## Abbreviations

Abstn: Abstain
CD: Condom use
GDHS: Ghana Demographic and Health Survey
GLSS: Ghana Living Standards Survey
GSS: Ghana Statistical Service
HIV/AIDS: Human immunodeficiency virus/Acquired immunodeficiency syndrome
ref: Reference
STI/D: Sexually Transmitted Infection/Disease
UNAIDS: United Nations Programme on HIV/AIDS
WHO: World Health Organisation

## References

[1] United Nations. Progress towards the Sustainable Development Goals. Report of the Secretary-General, United Nations Economic and Social Council. 2017.

[2] United Nations. Transforming our world: the 2030 Agenda for Sustainable Development". United Nations -Sustainable Development knowledge platform. [cited 13th September, 2022]. Available from https://www.un.org/sustainabledevelopment/developmentagenda/. 2015.

[3] World Health Organization. HIV [fact sheet]. [cited 13th September, 2022]. https://www.who. int/news-room/fact-sheets/detail/hiv-aids. 2021.

[4] Statista. HIV/AIDS worldwide. [cited 2022 Aug. 17]. Available from: https://www.statista.com/topics/773/hiv-aids-worldwide/. 2021.

[5] Ghana Demographic and Health Survey (GDHS). Ghana. https://dhsprogram.com/pubs/pdf/SR224/SR224.pdf. 2014.

[6] Nketiah-Amponsah E, Abubakari M, Baffour PT (2019). Effect of HIV/AIDS on economic growth in sub-Saharan Africa: recent evidence. Int Adv Econ Res.

[7] Ghana AIDS Commission: 90–90–90 Programme, vol. Ghana; 2021.

[8] Katey D, Addo AA (2022). HIV renaissance in Ghana: an opinion piece on further measures to address HIV among Ghanaian youth. Afr J AIDS Res.

[9] Fuchs VR. Introduction to" Economic Aspects of Health". InEconomic aspects of health 1982 Jan 1 (pp. 1–12). University of Chicago Press.

[10] Holt CA, Laury SK (2002). Risk aversion and incentive effects. Am Econ Rev.

[11] Anderson LR, Mellor JM (2008). Predicting health behaviors with an experimental measure of risk preference. J Health Econ.

[12] de Oliveira AC, Leonard TC, Shuval K, Skinner CS, Eckel C, Murdoch JC (2016). Economic preferences and obesity among a low-income African American community. J Econ Behav Org.

[13] Herberholz C (2020). Risk attitude, time preference and health behaviours in the Bangkok Metropolitan Area. J Behav Exp Econ.

[14] World Health Organization (WHO) Adolescent and young adult health.. [cited 13th Sept, 2022]. Accessed 30^th^ Aug 2020.

[15] Kasahun AW, Yitayal M, Girum T, Mohammed B. Risky sexual behavior and associated factors among high school students in Gondar City, Northwest Ethiopia. Int J Public Health Sci. 2017;6(3):257–65.

[16] Blais AR, Weber EU (2006). A domain-specific risk-taking (DOSPERT) scale for adult populations. Judgm Decis Mak.

[17] Markowitz H (1952). Portfolio Selection. J Financ.

[18] Kahneman D, Tversky A. Prospect Theory: An Analysis of Decision under Risk. Econometrica. 1979;47(2):263–92 Available from: https://www.jstor.org/stable/1914185.

[19] Binswanger HP (1981). Attitudes toward risk: Theoretical implications of an experiment in rural India. Econ J.

[20] Grossman M (1972). On the concept of health capital and the demand for health. J Polit Econ.

[21] van der Pol M, Ruggeri M (2008). Is risk attitude outcome specific within the health domain?. J Health Econ.

[22] Lawless L, Drichoutis AC, Nayga RM (2013). Time preferences and health behaviour: a review. Agric Food Econ.

[23] Dardanoni V, Wagstaff A (1990). Uncertainty and the demand for medical care. J Health Econ.

[24] Pfeifer C. A note on smoking behavior and health risk taking. Nordic Journal of Health Economics. 2012;1(2):135–51.

[25] Lépine A, Treibich C (2020). Risk aversion and HIV/AIDS: Evidence from Senegalese female sex workers. Soc Sci Med.

[26] Galizzi MM, Miraldo M (2017). Are you what you eat? Healthy behaviour and risk preferences. BE J Econ Anal Poli.

[27] Weinstein ND, Kwitel A, McCaul KD, Magnan RE, Gerrard M, Gibbons FX (2007). Risk perceptions: assessment and relationship to influenza vaccination. Health Psychol.

[28] Weller SC, Davis‐Beaty K, Cochrane HIV/AIDS Group. Condom effectiveness in reducing heterosexual HIV transmission. Cochrane database of systematic reviews. 1996;2012(3):1–7. 10.1002/14651858.CD003255 PMC8407100 11687062

[29] Maticka-Tyndale E (2012). Condoms in sub-Saharan Africa. Sex Health.

[30] Ghys PD, Diallo MO, Ettiègne-Traoré V, Kalé K, Tawil O, Caraël M, Traoré M, Mah-Bi G, De Cock KM, Wiktor SZ, Laga M. Increase in condom use and decline in HIV and sexually transmitted diseases among female sex workers in Abidjan, Cote d’Ivoire, 1991–1998. AIDS. 2002;16(2):251–8. 10.1097/00002030-200201250-00015 11807310

[31] Smith DK, Herbst JH, Zhang X, Rose CE (2015). Condom effectiveness for HIV prevention by consistency of use among men who have sex with men in the United States. JAIDS J Acquir Immune Defic Syndr.

[32] Government of Ghana. Ghana Population Stabilisation Report. 2011. Available from: http://www.populationcommunication.com/Medias/Ghana_report.pdf. cited 2023 Mar 14.

[33] Appiah-Agyekum NN, Kayi EA. Students’ perceptions of contraceptives in University of Ghana. J Family Reprod Health. 2013;7(1):39. PMC4064744 24971101

[34] Ghana Statistical Service. Demographic and health survey. Retrieved from https://dhsprogram.com/pubs/pdf/FR307/FR307.pdf. 2014.

[35] Karim AM, Magnani RJ, Morgan GT, Bond KC (2003). Reproductive health risk and protective factors among unmarried youth in Ghana. Int Fam Plan Perspect.

[36] Baah-Odoom D, Riley G (2012). Expanding the theory of planned behaviour: The influence of personal norms on condom use amongst young people in Ghana. J Soc Sci Public Policy..

[37] Bosompra K (2001). Determinants of condom use intentions of university students in Ghana: An application of the theory of reasoned action. Soc Sci Med.

[38] Barsky RB, Juster FT, Kimball MS, Shapiro MD (1997). Preference parameters and behavioral heterogeneity: An experimental approach in the health and retirement study. Q J Econ.

[39] Love DA, Smith PA (2010). Does health affect portfolio choice?. Health Econ.

[40] Fan E, Zhao R (2009). Health status and portfolio choice: Causality or heterogeneity?. J Bank Finance.

[41] Coile C, Milligan K (2009). How household portfolios evolve after retirement: The effect of aging and health shocks. Rev Income Wealth.

[42] Cardak BA, Wilkins R (2009). The determinants of household risky asset holdings: Australian evidence on background risk and other factors. J Bank Finance.

[43] Conner M, Norman P (2005). Predicting Health Behaviour: Research and Practice with Social Cognition Models.

[44] Weber EU, Blais AR, Betz NE (2002). A domain-specific risk-attitude scale: Measuring risk perceptions and risk behaviors. J Behav Decis Mak.

[45] Arrow KJ (1971). The Theory of Risk Aversion Chapter 3 in Essays in the Theory of Risk Bearing.

[46] Pratt JW (1964). Risk Aversion in the Small and in the Large. Econometrica.

[47] Eckel CC, Grossman PJ (2008). Forecasting risk attitudes: An experimental study using actual and forecast gamble choices. J Econ Behav Organ.

[48] Frempong RB, Stadelmann D (2021). Risk preference and child labor: Econometric evidence. Rev Dev Econ.

[49] Adjei-Mantey K, Horioka CY (2022). Determinants of health insurance enrollment and health expenditure in Ghana: An empirical analysis. Rev Econ Household.

[50] Adjei-Mantey K, Takeuchi K (2023). Risk aversion and cleaner cooking fuel choice: an empirical study in Ghana. Environ Dev Econ.

[51] Sutter M, Kocher MG, Glätzle-Rützler D, Trautmann ST. Impatience and uncertainty: Experimental decisions predict adolescents’ field behavior. Am Econ Rev. 2013;103(1):510–31.

[52] Leonard T, Shuval K, De Oliveira A, Skinner CS, Eckel C, Murdoch JC (2013). Health behavior and behavioral economics: economic preferences and physical activity stages of change in a low-income African-American community. Am J Health Promot.

[53] Mussio, I. Three Essays on Health, Risk and Behavio. Doctoral Dissertations. 1467. 2018 10.7275/12700436/scholarworks.umass.edu/dissertations_2/1467.

